# Future Directions for Personality Research: Contributing New Insights to the Understanding of Animal Behavior

**DOI:** 10.3390/ani9050240

**Published:** 2019-05-15

**Authors:** Vanessa Wilson, Anja Guenther, Øyvind Øverli, Martin W. Seltmann, Drew Altschul

**Affiliations:** 1 Cognitive Ethology Laboratory, German Primate Center, Kellnerweg 4, 37077 Göttingen, Germany; 2 Johann-Friedrich-Blumenbach Institute for Zoology and Anthropology, Georg-August-University, 37077 Göttingen, Germany; 3 Leibniz ScienceCampus Primate Cognition, 37077 Göttingen, Germany; 4 Department of Evolutionary Genetics, Max Planck Institute for Evolutionary Biology, 24306 Plön, Germany; guenther@evolbio.mpg.de; 5 Department of Food Safety and Infection Biology, Faculty of Veterinary Medicine, Norwegian University of Life Sciences, NO-0508 Oslo, Norway; oyvind.overli@nmbu.no; 6 Department of Biology, University of Turku, 20014 Turku, Finland; martin.seltmann@utu.fi; 7 Department of Psychology, The University of Edinburgh, Edinburgh EH8 9JZ, UK; Drew.Altschul@ed.ac.uk; 8 Center for Cognitive Ageing and Cognitive Epidemiology, The University of Edinburgh, Edinburgh EH8 9JZ, UK; 9 Scottish Primate Research Group

**Keywords:** individual differences, personality, developmental plasticity, fitness, longevity, animal welfare, stress coping, wellbeing, endocrinology

## Abstract

**Simple Summary:**

Personality—i.e., individual differences in behavior and emotion—is increasingly being recognized as important in animal research. Whilst numerous studies highlight behavioral variation across a diverse range of species, an understanding of what drives this variation, and how it is maintained, is still limited. Moreover, applying the study of individual differences to issues such as stress coping and health outcomes could be hugely beneficial to animal welfare. We discuss these topics, along with promising avenues for future personality research, which should benefit a broader understanding of animal behavior.

**Abstract:**

As part of the European Conference on Behavioral Biology 2018, we organized a symposium entitled, “*Animal personality: providing new insights into behavior?*” The aims of this symposium were to address current research in the personality field, spanning both behavioral ecology and psychology, to highlight the future directions for this research, and to consider whether differential approaches to studying behavior contribute something new to the understanding of animal behavior. In this paper, we discuss the study of endocrinology and ontogeny in understanding how behavioral variation is generated and maintained, despite selection pressures assumed to reduce this variation. We consider the potential mechanisms that could link certain traits to fitness outcomes through longevity and cognition. We also address the role of individual differences in stress coping, mortality, and health risk, and how the study of these relationships could be applied to improve animal welfare. From the insights provided by these topics, we assert that studying individual differences through the lens of personality has provided new directions in behavioral research, and we encourage further research in these directions, across this interdisciplinary field.

## 1. Introduction

The study of individual differences is now wide spread across both ecology and psychology. The importance of considering individual differences in the study of behavior [1,2,3], cognition [4,5], development [6,7], stress responses [8,9,10], fitness and life history outcomes [11,12], and wellbeing [13,14,15] has been well established. Yet, the field of personality which has sprung up amidst this research is not short of critics. Indeed, whether the field of animal personality can provide any new insights for the study of behavioral ecology was addressed by Beekman & Jordan [16], who argued that behavioral variation may occur as a result of environmental and genetic effects, or in a life-history trade-off, which does not require the use of the term ‘personality’. Whilst it is true that behavioral variation has long been acknowledged by behavioral ecologists, often it has been thought of as the raw material for natural selection, rather than the product [17,18,19]. That such variation exists is not under debate; rather, we ought to ask: How is such variation generated, and maintained, in the face of natural selection? And what underlying mechanisms drive this variation? The field of animal personality is beginning to address these questions by examining developmental plasticity and epigenetic effects [20,21,22], as well as assessing drivers of cognitive variation [5,23,24].

To highlight these recent advances in the study of individual differences, and the multi-faceted, interdisciplinary approaches that stretch beyond the fields of either behavioral ecology or psychology, we organized a symposium at the European Conference on Behavioral Biology 2018 entitled, “*Animal personality: providing new insights into behavior?*”. The goals of this symposium were twofold. First, we aimed to discuss causal approaches to understanding personality, such as looking at endocrinological effects and life history outcomes associated with particular traits, and in what ways personality may explain variation in cognition. Our second purpose was to highlight applications of personality research. In this respect, we wished to address how studying personality allows us to understand individual differences in stress coping, mortality, and health risk [10,25,26,27] and can be applied to the improvement of animal welfare and captive management at the individual level [14]. In the following sections, by addressing studies in wild, captive, and domesticated species, we highlight not only the research, theory, and approaches presented at the symposium, but also the future directions for this research. In doing so, we aim to demonstrate that the study of individual differences and the field of animal personality do, as a whole, contribute new insights into animal behavior.

## 2. Underlying Mechanisms of Individual Variation

Consistent individual differences in behavior, often labeled as personality [28], arise due to genetic effects [29,30,31] or as a result of irreversible developmental plasticity [6,32,33,34]. Developmental plasticity allows adjustment of the phenotype to environmental conditions experienced in early life, often with long-lasting effects that even remain constant in adulthood. Environmental conditions may act directly on the developing phenotype through developmental constraints. Poor nutrition in early larval stages for example reduces risk-taking behaviors in captive-reared fire salamanders (*Salamandra salamandra*), mediated by restricted growth and delayed metamorphosis [35]. Alternatively, environmental conditions may be mediated by parental effects, i.e., effects parents may have on the phenotype of their offspring over and above direct genetic transmission [36]. The male offspring of wild guinea pig (*Cavia aperea*) mothers that experience an unstable social environment during pregnancy and lactation, show reduced aggressiveness and enhanced anxiety-like behavior, compared to sons of mothers experiencing a stable social environment [37]. Likewise, the season into which offspring of wild guinea pigs are born shapes the personality type in adulthood, with animals born early in the breeding season showing more risk-taking and less fearful behavior [21]. Parental effects may be adaptive if they result in a phenotype that is better fitted to the environment the individual is likely to experience as an adult [38,39]. Developmental plasticity is particularly strong during early ontogeny and generally decreases with age [40]. Therefore, especially early sensitivity to environmental conditions during specific sensitive time windows in development may adaptively adjust personality [41].

These sensitive windows are short periods of time in which plasticity is heightened because of major changes in the physiology and ecological or social niche of the individual. Coming back to the examples of wild guinea pigs, both the physiology and ecological niche change around the timing of maturation. When becoming sexually mature, young males often leave their natal groups and habitats to disperse and roam around until they are strong enough to establish their own territory [42]. These changes in their ecological niche are accompanied by a steep increase in testosterone, exploration and risk-taking behavior [43], which are much less present in females of this species [21]. Males which stay in their natal groups have to integrate into the social hierarchy and exhibit a queuing strategy [44]. These males show reduced aggressiveness and enhanced fearful behavior. Especially for social species, the ecological and social niche are often inseparable, while certain ecological characteristics may play a more important role for non-social species.

The pre- and early postnatal phase, and recently, the period of adolescence, have been identified as sensitive windows with the potential to alter behavioral phenotypes (i.e., mean levels of behavior), behavioral consistency (i.e., repeatability) and even correlations among traits (i.e., behavioral syndromes) (see for example [45,46,47,48,49,50,51,52]). For example, harshness/stressfulness, predictability, and social aspects of the early environment have repeatedly been found to affect behaviors such as stress coping, anxiety and suites of social behaviors, including aggressiveness or gregariousness [53,54,55]. Harsh environmental conditions may be represented, e.g., by predation pressure. Lab-reared *Poecilia reticulata* as well as wild-caught *Brachyrhaphis episcopi* poeciliid fish adjust their adult behavioral phenotype to the level of predation experienced in early life by increasing exploration behavior and adjusting levels of activity and risk-taking behaviors [56,57]. Harshness or stressfulness of the environment may also be experienced by low food quality or quantity. Captive field crickets (*Gryllus bimaculatus)* reared on high quality food were more aggressive in adulthood [48]. Other environmental characteristics such as temperature or pesticide exposure may also contribute to the stressfulness or harshness of the environment and have been shown to exert long-lasting effects on behavioral phenotypes [58,59]. The stability and predictability of the environment was also shown to influence the behavioral phenotype. Captive-bred eastern mosquito fish (*Gambusia holbrooki*) developed a bolder and more active phenotype when sourced from a more predictable habitat compared to fish from an unpredictable habitat [60],

The proximate mechanisms by which genes and environmental cues shape consistent individual differences during development are still poorly understood, although theoretical studies have suggested various state variables as driving forces for individual differences [61,62,63]. Metabolic rate and circulating hormone levels were found to covary significantly albeit with relatively small effect sizes with personality traits [64]. Endocrinological candidate systems that could help unravel the driving forces of personality variation are the hypothalamus–pituitary–adrenal (HPA) and the hypothalamus–pituitary–gonadal (HPG)-axes, which show particular promise in vertebrates (reviewed in Reference [65]). Acting through steroid hormones (mainly cortisol/corticosterone and testosterone), the HPA- and HPG-axes mediate diverse functions related to energy balance and reproduction, thereby giving rise to differences in behaviors related to resource acquisition and allocation (e.g., risk-taking, exploration, agonistic interactions). Glucocorticoids coordinate responses to changing environmental conditions by initiating physiological and behavioral stress responses [66,67]. The sex steroid testosterone is a key hormone with regard to sexual behavior and secondary sexual ornaments, and is involved in the expression of behaviors broadly related to reproduction, e.g., parental investment/care and agonistic interactions [68]. Importantly, steroids have been identified as the main driving forces for developmental plasticity by exerting long-lasting, pleiotropic effects during sensitive time windows in ontogeny [69].

Recently, hormones coordinating growth and related behaviors such as foraging and risk-taking have come into focus as possible mediators for personality differences. Juvenile hormones are responsible for a number of organizational functions in invertebrates, regulating growth, development and investment into reproduction, including behaviors [70,71,72]. For example, the juvenile hormone is largely responsible for mediating sex-differences in parental care between sexes in insects [72] and higher titers of it increase reproductive behaviors such as male–male competition and secondary sexual signaling [73]. In vertebrates, thyroid hormones serve many similar functions [74], but the incorporation of these hormones into personality research has yet to be done [75].

Neuroendocrinological systems generally exert pleiotropic effects that affect many traits such as behavioral, physiological, and reproductive traits at the same time. To date, an unanswered question in this respect is whether such pleiotropic effects of the neuroendocrinological system can also explain the frequently found associations between personality and cognitive traits [76]. Investigating the causes and consequences of personality–cognitive covariation is a recently ascending research field [77,78]. Another possibility is that personality and cognitive traits share a more direct, facilitating or inhibiting, relationship. Specific personality characteristics for example may facilitate or constrain the rate at which environmental cues are encountered, or rules about the environment are learned [24,78]. Plausible scenarios for such relationships would be if bold, curious, or explorative individuals learn a particular cognitive task faster, either because they are more likely to encounter the task, they pay more attention to changes in their surroundings, they store information faster, have lower decision thresholds for association formation, or memorize associations more readily. For example, domesticated guinea pigs (*Cavia porcellus*) which approach an unknown object quickly have been found to learn to associate a food reward with a symbol faster than shyer conspecifics, while the shyer animals were faster to reverse the association in a reversal learning task, showing that they paid more attention to slight changes in the test setup. Bold animals were also better in solving novel problems explained by their shorter latencies and longer interaction times with the test setups [79,80]. So far, no studies have addressed the mechanistic relationship between personality–cognitive covariation, although such relationships will likely have important ecological and evolutionary consequences.

Evidence from neuroanatomical studies, however, suggests that a relationship between brain structure and personality could underlie links to cognition. For example, grey matter volume in the anterior cingulate cortex, which has been implicated in attention and emotion regulation, was linked to measures of higher openness and extraversion, and lower dominance in captive chimpanzees (*Pan troglodytes*) [81]. Further links to the brain have been found in humans: cortical thickness [82], network efficiency [83], white matter microstructure [84], as well as total [85] and regional [86] brain volume are all associated with various personality traits. It has also been suggested that neural plasticity, mediated through hormonal changes in response to the environment, could be linked to behavioral flexibility [87]. Examination of the neuroanatomy and neural plasticity underlying behavior therefore could be a promising avenue of research in understanding personality–cognition covariation.

## 3. Longevity’s Insights into Fitness and Health

Responses to the environment, such as those reflected by novel object tests but also in measures of cognitively driven behaviors like innovation [88], can be linked to fitness [12,89], which can itself be assessed through proxy measures such as mate choice or survival. For example, faster reaction time to predation stimuli has been linked to better survival in the wild African striped mouse (*Rhabdomys pumilio*) [90]. However, ‘good’ performance does not necessarily predict higher fitness. In captive reared pheasants (*Phasianus colchicus*), for example, individuals that were slow at reversal learning, i.e., poorer performers, had higher survival upon release [91]. To understand the nature of the varying relationships between individual psychological differences (i.e., individual cognitive and behavioral differences) and fitness, it is necessary to consider definitions of ‘fitness’ in more detail.

Life history theory posits that there is a continuum of viable strategies for increasing individual fitness [92]. At one end of the continuum are fast life history strategies, characterized by both early and frequent reproductive attempts, rapid deterioration of physical health, and shorter lifespan. At the other end are slow life history strategies, characterized by less frequent reproduction that extends into the animals’ longer lives. An animal’s longevity can thus tell us much about its life history strategy.

Differences in life history strategies are a possible explanation for the existence of spectrums of consistent suites of behaviors, or in other words, personality traits [93,94]. Reviews of studies on animal boldness, exploration, and aggression have supported this, although meta-analytic evidence has been mixed [12,95,96]. Longer life and greater reproductive success are two sides of the same coin, so individuals who live longer should also have lower reproductive success in order for there to be a trade-off. For example, bolder animals die younger but have greater reproductive success [12]. On the other hand, more aggression was associated with greater reproductive success, but not longevity, and more exploratory individuals lived longer, but did not appear to reproduce less.

Reproductive success is often considered to be the most important indicator of fitness, but other indicators (e.g., size at birth and maturity, reproductive scheduling) must be considered to understand the full complement of evolutionary forces that shape animal behavior. Longevity is particularly important in the case of longer lived animals, such as primates in captivity [97], wild and captive elephants (both *Elephas maximus* and *Loxodonta africanus*) [98,99], wild and captive turtles [100], and captive-bred lobsters *(Homarus americanus)* [101]. During these animals’ long lives, cumulative effects, of, for example, investment in offspring and other relatives, increases individual fitness [102]. Particularly in primates, studies of longevity are not uncommon [103,104], and several large studies of personality and longevity demonstrate meaningful associations between personality traits, particularly social personality traits, and longevity [25,27].

There are two major limitations on the strength of longevity studies: sample size and follow-up time. However, as the field of animal behavior advances, both of these limitations will become less concerning. The first, sample size, is currently a major issue across the biological and behavioral sciences [105,106,107,108], and grappling with this problem must be a part of any progression in the field of behavioral biology. Studies of longevity and individual differences are as susceptible to errors induced by small sample sizes as any other behavioral biology study, possibly more so. Associations between personality and life history traits are often small [12,25], so while longevity can tell us much about the evolution of individual differences, researchers need to consider power and where possible, carry out a priori power analyses.

The second limitation in longevity studies comes from follow-up time, which is directly linked to the age of an initial survey. As animal personality research has developed and proliferated, more studies and study sites have sprung up. In contrast to reproductive output, which requires careful tracking of individuals and groups, longevity data are less laborious to collect, at least in large, long-lived animals. Many existing studies might be re-analyzed with the addition of longevity data. Power in survival analyses is largely derived from the number of events, otherwise known as deaths in longevity research. Thus, the longer the follow-up time from initial personality measurement, the more animals that will have died, and the greater the power of the study.

Longevity and its correlates also allow us to ask deeper questions about the health processes of organisms. For example, low agreeableness chimpanzees are more likely to die younger [25], but what are the specific processes linking agreeableness and mortality? Associations between personality and longevity need not be driven through the same mechanism across different dimensions or between species. For example, in captive raised guppies (*Poecilia reticulata*), boldness is associated with a stronger tendency to inspect predators, which directly increases the probability of death [109]. On the other hand, in wild-caught aquatic snails (*Radix balthica*), higher boldness is linked to more protective shell shapes, neutralizing at least some of the negative consequences of living boldly [110]. However, making stronger shells is energetically costly, and possibly reduces the lifespan through these costs.

Similarly, different possible mechanisms might be viable within species as well. The less agreeable chimpanzees mentioned in the previous paragraph are more aggressive [111], and more aggression is associated with more injury and death, but it is also associated with a higher metabolic rate [94]. Increased metabolism results in more free radical, reactive oxygen species being produced within an organism at the cellular level, which can lead to oxidative damage, and accelerated biological aging. In this way, personality might be indirectly linked to reduced lifespan.

Through this lens, the applied utility of personality and longevity studies becomes apparent. If a more aggressive individual is more often injured and/or aging at an increased rate, they will be more likely to experience comorbid health issues [112,113] that could impact their quality of life. With the knowledge that individuals with particular personalities are more at risk for health problems, captive care facilities could devote additional resources to at-risk individuals. Altogether, our understanding of longevity and personality is relevant to breeding efforts, particularly relevant in threatened species, and preservation of individual welfare.

## 4. The Concept of Stress Coping Styles and Its Relation to Animal Welfare

Welfare assessment is becoming increasingly important in current day zoological and agricultural research [13,114,115,116]. In zoos, this issue is particularly vital to the increasing number of captive breeding programs, which aim to conserve natural behaviors amongst captive, threatened species whilst simultaneously trying to provide these animals with adjustment to a restricted environment where they may live their whole lives [14]. In farming, welfare considerations are increasing in response to growing consumer demand and intensification of livestock production, which simultaneously contributes to concern for the health and wellbeing of animals bred for their products. This has made welfare research a pertinent topic in the animal sciences.

Regardless of strict definition, the concept of animal welfare is based on the belief that animals have subjective emotions and have the ability to feel pleasure and suffer [117]. With regards to our ability to ‘measure’ different aspects of animal welfare, this view is in contrast to a positivistic and behavioristic scientific view, where science deals only with the material world, and not with emotion. However, even emergent phenomena such as emotion and cognitive processes are rooted in the material world, specifically in measurable physiological processes creating the communication between networked nerve cells. In the following, we outline the argument that behavioral and physiological adjustments in response to stressful and aversive situations, as well as associated cognitive/emotional processes, should be seen fundamentally as adaptive responses to changing environments. From this stance, we develop a framework for how to include individual phenotypic variation in physiological and behavioral personality traits in the study of animal welfare.

A long term consensus definition of ‘stress’ was very precisely summarized by Moberg [118], when stating that ‘stress’ is “the biological response elicited when an individual perceives a threat to its homeostasis”. This definition encompasses the notion that psychological processes associated with how an individual assesses a given situation might be equally important as the actual physical challenge in determining the severity of the stress response [119]. In animals as well as humans, repeated or prolonged exposure to stress can potentially compromise the physiological and psychological basis for health and welfare. Under the above definition of stress, individual variation in responsiveness can be of particular importance for animal welfare, since conditions that are well tolerated by some individuals may be detrimental to others. For example, in farmed Atlantic salmon (*Salmo salar*), fish that avoid aggressive interactions at fixed feeding sites exhibit slower growth rates than those that compete for space near the feeder, which can result in the loss of condition and compromised welfare [120].

An influential paper by Korte et al. [121] however, advised on a potential pitfall regarding a direct relationship between homeostasis and animal welfare: the concept of homeostasis implies that the purpose of physiological regulation is to maintain physiological parameters at pre-defined settings. However, successful acclimation to changing environments involves significant resetting of neural, endocrine, and immune mechanisms (i.e., allostasis, or “stability through change” [112,122,123]). This implies that many physiological and behavioral processes commonly labeled “stress responses” rather could be seen as adaptive adjustments, through which animals respond to both predictable and unpredictable events [124,125], which is further subject to individual phenotypic variation in the form of stress response and coping style [8,9].

Various terms are used to categorize individuals employing consistently different reaction norms in response to changes in the environment, but when physiological correlates of consistent behavioral patterns are concerned, animals are commonly classified as either “reactive” or “proactive” based on their distribution along a shy–bold continuum (for reviews, see [8,10,126]). Stress coping styles can be thus defined by the set of behavioral and physiological responses to stress that are consistently employed by one individual across unrelated and temporally separated situations [127]. Reactive individuals are characterized by having a high post-stress corticosteroid production and low activity of the sympathetic adrenal-medullar system. They display low levels of aggression, “freeze and hide” behavior, high behavioral flexibility, and low risk taking. By contrast, bold and proactive individuals are characterized by low corticosteroid responses and high sympathetic activity. These individuals tend to employ a “fight or flight” strategy when stressed, are aggressive, rigid, and routine forming and generally display high-risk behavior. Assessment of these characteristics has been employed across a range of wild, laboratory, and farmed species, such as rats (*Rattus norvegicus*) and other small mammals, pigs (*Sus scrofa domesticus*), cows (*Bos taurus*), rainbow trout (*Onchorhynchus mykiss*), and flatfish (e.g., halibut, *Hippoglossus hippoglossus*, and sole, *Solea senegalensis*). These studies reveal similarities in copying style traits across these different species, indicating evolutionarily conserved traits for coping mechanisms in vertebrates [8,126,127,128,129].

From an evolutionary perspective, typical proactive responses such as aggression and active avoidance are considered likely to increase fitness when stressors are mild, predictable, and of short duration [130]. Under chronic, severe, or unpredictable stress, the organism is better off by reducing risk taking and conserving energy by passive behavioral responses. Reactive individuals are thus thought to perform better in unstable environments. The functionalist approach to emotions [131] holds that emotions have evolved for a particular function, such as inducing appropriate behavioral responses to potentially dangerous stimuli. Cognitive changes and emotional distress can thus be considered a likely correlate of passive coping. In this view, welfare is the animal’s overall subjective experience, a real phenomenon emerging from the brain’s motivational and cognitive systems, developed through evolution as an integrated part of the survival mechanisms in animals with advanced central neural systems. Furthermore, emotions or affective states are evolved adaptations that play important roles in causing behavior. In this concept, impaired welfare arises only when “allostatic overload” occurs from chronic, unpredictable, or uncontrollable conditions which do not merit successful allostatic adjustment [132,133,134]. Therefore, individual variation in the threshold for when a challenge becomes inhibiting rather than stimulatory, i.e., coping style or personality, is likely correlated to the individual’s subjective experience of welfare in a given situation.

## 5. Applying Personality to Improve the Welfare of Captive Animals

In humans, personality is an important driver for wellbeing and health [135,136]. It is therefore not surprising that personality is considered an important factor when decisions have to be made about human–human interactions and well-being. For example, companies regularly use personality tests in job interviews to check an applicant’s suitability, and they play a strong role in relationship counselling, school psychology, and occupational health and safety.

Accepting the importance of personality for wellbeing in humans prompts an interesting question: Why is the importance of personality for the wellbeing of non-human animals not more recognized? In addition to considering welfare for animals housed in zoos and those used in food production (see previous section), animal welfare plays an important role for working animals (such as race horses, military dogs, and therapy animals [15,137,138]), lab animals [139] and also for companion animals [140]. In all these examples, human–animal interactions occur, and it is the human’s responsibility as caretaker, pet-owner, or work colleague to be aware of the welfare needs of the animals they interact with. Why, therefore, has personality been considered less relevant for welfare in non-human species?

Many studies have looked at the personality of zoo animals, working animals, and pets [140,141,142], with several studies examining the potential link between personality and wellbeing of animals [143,144,145]. Typically, authors recommend assessing personality because of its potential for improving wellbeing [144], yet less work is done on how to implement wellbeing improvements in non-human animals. Some studies show that individuals with certain personality traits, or combinations of traits, [146,147] have greater reproductive success. For instance, according to findings of a study on captive giant pandas (*Ailuropoda melanoleuca*), breeding managers should try to mate low-aggressive females with high-aggressive males or mate pairs with similar scores on fearfulness [148]. The link between mating partner personality and reproductive success is, however, species- and environment-specific, and is more a management concern than a welfare concern, since welfare is not just successful reproduction. Additionally, if captive populations are being managed with the purpose of re-introducing individuals to the wild, the trade-off between welfare and reproduction has to be considered: whilst some behaviors or personality traits may improve welfare in captivity, the same traits might be much less beneficial in the wild [144].

Several critical issues arise when considering the application of personality for improving wellbeing in non-human animals. First, we have to know how an individual animal experiences a certain situation, rather than how humans expect the animal to experience it. Second, as with the link between personality and reproduction, the link between personality and welfare is likely species-specific. We each have a subjective experience of fear, and thus we could each expect someone else to have that same experience [149]. As with different stress coping styles, individuals with different personalities might experience fear very differently. If an individual in a captive environment is for example consistently more fearful than its cohabitants, its welfare and its capability to cope with this environment could be impaired. Therefore, it would be of great benefit for animal welfare and management if we strive to improve our understanding of the link between personality and wellbeing in many species.

It is clear that well-designed, species-specific questionnaires for captive animals which allow for comparisons between similar, long-lived species housed in captivity, need to be developed. Studies then have to collect data with those questionnaires to assess the basic personality components of a species and also their (subjective) wellbeing. These findings will need to be validated by correlating questionnaire findings with behavioral observations and physiological measures of wellbeing and health so we can ensure the biological relevance of the questionnaire results. We must also consider the right time to perform a questionnaire—should personality be assessed already before the animal reaches maturity, when it is still developing? Or only later in life, when its personality has developed but its welfare may already be seriously compromised? It is also important to consider the personality of the caretakers or owners, as this can interact with the animal’s personality and wellbeing [150]. New questions then arise: how much extra time and money would it cost to score at least every long-lived zoo or working animal or pet with higher cognitive abilities for its personality and wellbeing? Would society be interested or willing to contribute to these costs? Is there public awareness about the scientific concepts, causes, and consequences of animal personality and animal wellbeing? Should society put so much effort into understanding animal personality and improving the welfare of animals in captivity? From a purely ethical standpoint, the answer should be yes. However, ethics are not the only factor in question, and we have to consider the financial and logistical aspects too. Finding solutions to manage the trade-offs between resources and the ethical ideal should be a top priority.

To address these issues in context, we consider recent research on the Asian elephant, which aims to use handlers’ knowledge of their personality to improve and guarantee the wellbeing of a semi-captive population of logging elephants in Myanmar [151,152,153]. Asian elephants are often used for work or religious ceremonies throughout their range countries, and Myanmar has the largest captive population of Asian elephants in the world. The Myanmar Timber Enterprise (MTE) employs over half of that population for work in the timber industry as transport and draught animals, with strictly regulated working hours. Currently, MTE elephants receive regular veterinary care, and each elephant has a logbook in which vets and mahouts (elephant head riders) note down the elephant’s life events in detail. The relationship between a mahout and his elephant can often last a lifetime and therefore mahouts gain excellent knowledge about their elephants’ behaviors and needs.

The personality structure of these semi-captive elephants was recently assessed, through questionnaires to mahouts of 257 individual elephants. This study identified three personality factors in Asian elephants: attentiveness, sociability, and aggressiveness [151]. Attentiveness is related to the response of the elephant towards commands from mahouts and how it acts in its environment in general. While elephants that score high on Sociability seek closeness to other elephants and show positive social behaviors, elephants with high Aggressiveness scores on the other hand behave aggressively towards conspecifics, interfere in social interactions and displace other individuals. Generally, it has been shown that the knowledge and experience of caretakers is important to the assessment of animal welfare [144,154] and a call was made to utilize this valuable source of knowledge to increase the welfare of zoo animals [155]. Asian elephants are considered as endangered on the Red List of threatened species [156] and since captive populations can be an important source for wildlife conservation, it is important to utilize mahouts’ profound knowledge about the individual characteristics of their elephants to improve and guarantee the wellbeing of Myanmar’s working elephants.

Towards such an endeavor, and relatedly, to the study of other captive species and working animals, the following questions could provide valuable insight: (1) How do age, experience and personality of caretakers interact with the personality of the animals they care for? (2) In working animals, is it better if young and less experienced handlers are paired with animals that score higher on traits such as attentiveness, thereby reducing stress in both partners? (3) Are individuals which generally score higher on attentiveness or sociability less stressed? (4) Are working animals with certain personalities in certain work group compositions more stressed? (5) Are individuals scoring low on attentiveness attributes more prone to accidents? (6) Are individuals with certain personalities more prone to health problems in their working environment? (7) Are some personality types more suited for certain tasks? (8) Are some personality types more susceptible to soft training methods? Addressing these questions and ideas could provide us with helpful insights to keep working and captive animals healthy and, ultimately, happy.

## 6. Future Directions for Personality Research

As this discussion highlights, the study of individual differences may have far reaching consequences for the field of animal behavior. Understanding the underlying causal mechanisms of behavioral variation, such as the interplay between genetic and developmental factors or the links between endocrinology and consistent behavioral variation, should help us to understand how behavioral variation is generated, and maintained, in the face of natural selection. This moves beyond the basic acceptance that behavioral variation is a result of either environmental or genetic variation, and also moves beyond the assessment of behavior at fixed time points. To truly understand the occurrence and consistency of individual differences, they should be assessed across the lifespan, and should take into account early developmental windows such as the prenatal period [33,34,46,157].

The future for personality research is promising. Research has yet to fully address the mechanisms through which environmental effects shape differential development, or to what extent personality is shaped by later life experiences; similarly, the mechanisms linking personality to cognition and lifespan are poorly understood. In addressing these questions, we can begin to understand why some animals are better at solving problems than others, why some are more prone to stress, or why some individuals are more susceptible to early mortality or disease. Understanding the underlying mechanisms of fitness outcomes is fundamental if we wish to address issues such as wellbeing at the individual level. From an applied perspective, the adoption of coping style measures by animal care bodies could help to highlight indicators of compromised welfare and address individual welfare needs. Considering health outcomes, it could also be useful to determine whether certain traits consistently predict health outcomes across species, or whether personality health associations are species-specific. These considerations could be of particular importance to breeding programs and animal management, encouraging reduced mortality, and successful reproduction on an individual basis.

One limiting factor in answering these questions is sample size. Following the example of the ManyBabies [158] and ManyPrimates [159] projects, behavioral research could do well to counter the reproducibility and replication crises facing science, by pooling resources and setting up multi-lab collaborations, which would allow for standardized data collection with more appropriate sample sizes. Such collaborations could take decades to complete and may not be in the interest of researchers seeking quick gains to satisfy funding demands. However, as with large, longitudinal datasets such as the UK national longitudinal cohort studies [160,161,162,163] and even multi-generational datasets like the Cebu Longitudinal Health and Nutrition Study [164,165] and the US National Longitudinal Studies of Youth [166], this approach is likely more effective for research in the long term. In this discussion, we wished to highlight the new avenues of research that are opening up under the focused study of individual differences. We have exhibited not only some of the novel findings coming out of this field, but also the interdisciplinary nature that suggests promising future directions in the study of personality. In particular, we note the applied nature to this research, which provides not only insights into animal behavior, but has the potential to improve health, wellbeing, and longevity across species. We encourage other researchers to consider these approaches in future personality research.
